# Medical and Philosophical Intelligence

**Published:** 1816-02

**Authors:** 


### MEDICAL AND PHILOSOPHICAL INTELLIGENCE.

*To the Editors of the London Medical and Physical Journal.*

GENTLEMEN,

BELIEVING that it may be interesting to many of your readers, to see a correct statement of what passed on the 8th of December last, when the trial of the indictment of the King against Taunton was called on, I inclose a transcript from the short-hand writer's notes. I shall forbear making any observations on the manner of detailing this transaction in your last Number, and content myself with simply stating, that the account there given is extremely inaccurate. I am,

*Sun-court, Cornhill;* Your obedient servant,
*January* 19, 1816. CHARLES MURRAY.

*Court of King's Bench,*
*Friday, December* 8, 1815.

THE KING *v.* TAUNTON.

The list of the special jury were called, and only ten appeared.

*Mr. Attorney-general.*— "Your lordship will, I am sure, excuse my saying a word or two upon the course we are about to pursue. Your lordship has probably had an opportunity of casting your eye over the indictment, and of observing what is the subject of the prosecution. Those who think it is of infinite importance, that the plague of the small-pox should not infest the metropolis, as far as human beings can prevent it, have instituted this proceeding, in consequence of its having been alledged (and I will say no more at present than that it is alledged) that the defendant has been in the practice of bringing great numbers of persons to his house for the purpose of being inoculated, and of causing them to pass through the streets in all directions, and in every stage of the disease. My learned friend, Mr. Pollock, is for the defendant, and he states, that a learned friend of ours, his senior, is not here. Of that, I would say, that the absence of any gentleman while Mr. Pollock was here, was of no importance to the defendant. But he says that which is certainly much more important, that, since this prosecution, the defendant has given directions that this shall be prevented; and that being stated, I have no objection that this shall stand over, and I consent to its standing over, in the confidence that those who advise the defendant will enforce upon him the importance of that direction, which it is stated he has given, being attended to. God forbid that those who have the small-pox should not be attended in their own houses by any person they choose, but they must not be carried about the streets to the destruction of others. My friend will communicate to his client what has passed, and the understanding upon which this is allowed to stand over."

*Mr. Justice Bayley.*—" I hope that it is sufficiently notorious, that the causing persons to pass through the streets who may have that disorder upon them, although they are going for medical advice to some person in whom they have confidence, is an indictable offence; and, if that person, instead of attending them at their own houses as he might do, chooses to direct that they shall, from time to time, be brought or come to him, there is no question that he is liable to an indictment."

*Mr. Attorney-general.*—" The few sentences your lordship has pronounced now, are of the last importance to the community."

*Mr. Pollock.*—" Will your lordship excuse my stating, that as soon as it was known, from the judgment of the court in a former case, that this could not be done at the defendant's house, every time a patient has since attended him for the purpose of being inoculated, not only verbal directions, but likewise written directions have been given to the mother or the nurse, to take this child home, and not bring it out again till the disorder is removed; and by no means allow it to be with other children, so as to occasion infection."

*Mr. Justice Bayley.*—" And, in addition to that, Mr. Taunton should intimate, that he is ready to attend those persons at their own houses."

*Mr. Pollock.*—" I understand that is part of the notice, that he is willing to attend such patients at their own houses."

[We are much obliged to Mr. Murray for the above communication; by which it appears, that the law does not interfere with inoculation of small-pox, but with the exposure of patients under the disease, whether casually received or by inoculation. We do not see wherein the difference exists between our former report and the present, but a wish to shew our impartiality has induced us to insert the above.]

---

The following are the principal papers we have been able to select from the German and Flemish journals.

Dr. Kraft, at Runkel on the Lahn, describes a very singular appearance he beheld in the typhus castrensis, viz. *a blue nose:* this appearance occurs but seldom; and Dr. Kraft affirms, that amongst 300 of his private patients labouring under the typhus in 1813, neither he nor any of his colleagues had observed it; and in the Military Hospital at Runkel, amongst at least 700 patients, only seven or eight cases occurred.

In the Prussian Military Hospital, he saw two patients in whom this this singular appearance took place, and made the following observations on it. Some days after they had felt themselves ill, when loss of appetite, chilliness, vertigo, head-ach, and all the other symptoms of the typhus, had taken place, the tip of the nose began to turn red. The redness, about the same colour as in scarlatina, spread itself in the space of ten or twelve hours over the whole nose, assuming a paler tint on the cheeks and upper lip. The nose began to swell; the red colour became darker; a stronger fever, with all the symptoms of a more violent typhus, took place, succeeded by somnolency, and a sudden decline of the vital powers; the colour of the nose turned violet, and assumed at last a lead colour; the patient became insensible; and, though every possible exertion had been made for saving them, within about twenty-four or thirty-six hours from the beginning redness of the nose, inevitable death closed the catastrophe.

In one, the red colour of the nose gradually spread over both cheeks; the chin, throat, and breast down to the navel, then turned violet, and at last assumed the livid colour of mortification. According to the observations of the military physicians, there is no doubt that these patients afflicted with the red nose spread the miasma. The explanation of this appearance is difficult; but, with regard to the prognosis, the inference may with certainty be deduced.

Professor Loebel's *Observations upon the Benefit of Insolation in different Complaints, particularly in Cases of the Amaurosis.*— The physicians of former times cured different complaints by exposing the suffering parts to the action of the sun-beams, and practised this method particularly in disorders of the lymphatic system, such as different kinds of dropsy, and the gout. Professor Loebel has proved, in a dissertation, that insolation is with injustice neglected at present. According to his opinion, the effects of insolation upon the suffering organs are the following:

1. The warmth of the sun increases the activity of the lymphatic system, and of the vessels.
2. By the influx of light, the vital activity is roused and heightened both in the afflicted part and the whole body.
3. The development of oxygen, or vital air, caused by the action of the sun, also operates chemically upon the organization.

For these reasons, M. Loebel recommends insolation in the following cases:

1. In chronic anasarca, not founded upon any organic defect, where the extremities feel cold, and a general weakness and torpor appears in the lymphatic system, particularly in metastatic exanthema, after repelling the tinea capitis, the itch, or herpes, or after an ill-managed scarlatina.
2. In the chronic gout, particularly when all the organs are suffering by its long duration, when contractions, tumores ossium, and insupportable pains prevail, also where the gout leaves a partial palsy.

3. In

3. In all complaints attacking the tractus intestinorum, such as chronic spasms of the stomach, where weakness in the nervous system prevails, and in chronic diarrhœa; as also in the fluxus cœliacus and hepaticus, in chronic catarrh and chronic erysipelas.

4. In different forms of venereal complaints, in particular during the use of mercurials, when it serves to heighten and increase the effect upon the lymphatic system and the skin.

5. In diseases of the bones, tumores ossium, and in general or partial caries.

6. In subjects weakened by immoderate venery.

7. In nervous apoplexy, and palsy of single parts.

8. In the nervous gout of the head.

9. Against aphonie, when the incapacity of speaking is transitory, and not occasioned by the destruction of the organs.

10. In the marasmus senilis.

11. In the palsy of the lower extremities, particularly where the nervi crurales have suffered, and a state of inactivity and want of irritability prevails in them.

12. In amaurosis, from idiopatic causes, and from weakness of the retina or ciliary nerves, or when a palsied state of the optic nerve produces this complaint, or when it arises from a metastasis of gouty, venereal, or itchy matter.

Contra indications of the method of cure are the following:

*a.* In all diseases where an exalted irritability or plethora prevails, insolation must not be applied.

*b.* Neither in acute violent inflammations, general nor local; in affections of the lungs, which shew a disposition to inflammations, spitting of blood, or congestion.

*c.* Apoplexy, the nature of which consists in a congestion.

*d.* In hæmorrhages both of the active and passive kind, insolation is improper.

*e.* Insolation must absolutely be refrained from where the patient shews an idiosyncrasy or apathy against this method of cure, or by persons whose nerves, when in health, were found too sensible of the action of light or sun-beams, and who felt head-ach or vertigo on the slightest action of sun-shine.

*Manner of Application.*—1. This method of cure must not be applied in stormy or moist weather, or when east, west, and north winds prevail. Insolation requires calm days.

2. The patient during insolation must not sit or lie on the bare ground, but a leather skin must be placed under him, as was the custom of the Greek and Roman physicians.

3. Insolation must neither be applied on an empty stomach, nor directly after dinner; but, if the complaint requires the application during noon-tide, it is advisable to let the patient previously take a little food.

4. If insolation is to be performed on single suffering parts, the rest of the body must be covered with a white linen cloth, and only that part exposed on which the sun-beams are to act.

5. Insolation

5. Insolation must be adapted to the different cases: in one case half an hour or an hour is requisite, in others a period of some hours. Again, in some cases the rays of the morning sun, in others the most powerful rays of noon or afternoon, are necessary, according to circumstances and individuality. In complaints of the eyes, viz. in the amaurosis, Mr. Loebel advises to shut the eyelids, and to let the sun-beams act through half convex glass, placed upon the eyes thus shut.

Mr. Loebel gives the following directions for a particular machine which he calls a sun-bath. It consists of a box entirely constructed of panes of glass, about three feet long, nearly in the form of a hot-bed, the bottom to be of wood covered with sole-leather, the sides about three or four feet in height of panes of glass. In the upper part, an opening for the patient to put his head through; on one side a glass door by which to enter; and the bottom must be covered with very dry sand, or kitchen salt, about a quarter of a yard high. This box must then be exposed to the sun, so that the beams, thus more concentrated by the panes of glass, may produce a stronger effect upon the subject enclosed, for which reason there ought also to be small glass doors in the machine, that the degrees of heat may either be increased or lessened. The preference of this sun-bath to the usual method of insolation amongst the ancients consists in the more effectual application of the sun-beams upon the naked body, in proportion to the complaint, and according to the will of the physician; and, in case the perspiration of the patient takes place, the risk of catching cold is thereby absolutely prevented. The effect of the heat must also be far more powerful and concentrated than can be the case by pursuing the ancient method. Besides the ancient method cannot always be applied either in England or the northern parts of Germany, without risk to the patient, on account of the instability of the atmosphere; but the sun-bath recommended here may, with proper precaution, be applied more frequently and with greater confidence.

6. Finally, insolation must not be applied alone, but combined with those remedies adapted and prescribed for every form of sickness, as many cases require not only inward medicines, but also outward applications, such as frictions, &c.

Professor Loebel communicates the following remarkable cure of an amaurosis by applying local insolation.

J. S., a native of Dresden, forty years of age, had served from his nineteenth to his thirty-fifth year in the Saxon infantry, and amongst other excesses had profusely indulged in venery. When thirty-seven years old, he suffered much from the gout, but was restored. About two years after, he was seized with nervous apoplexy, which palsied his whole right side: he was also cured of this complaint, but it left a weakness in the organs of sight, which, in 1809, amounted to amaurosis in his right eye; by his left he saw, as he expressed himself, only as through a gauze. In 1810

he consulted Mr. Loebel, who, on close examination, was convinced that this amaurosis, the consequence of a complaint in the *nervi ciliares*, was connected with a weakness general to the whole organ. He therefore gave him a number of stimulating and nervous remedies internally and locally. Among the rest, very small doses of phosphorus. Under this management, the sight of the left eye was sensibly improved, and the gauze-like film disappeared; but the right eye, notwithstanding the inward and outward application of phosphorus, remained insensible, and the pupilla remained immoveably enlarged, and paralysed. He now resolved to apply local insolation, along with the use of the following prescription:

℞. Rad. Valer. Pulv. ʒijſs.

Cariophyl. Arom. ℈j.

Cortex Cinnam. ʒſs.

Spir. Vin. Gall. Opt. ℥iv.

To be taken a table-spoonful every two hours. For this purpose he fixed a silver wire round what is called a burning-glass, and, by means of ribbons fastened to each side, tied this convex glass upon the eye afflicted with the amaurose; then caused the rays of the meridian sun to operate through this glass, at first, only for half an hour. He directed the patient to shut his eye-lids during the insolation, and ordered the other parts of the head to be covered with a white linen cloth. The phosphoric infrictions were now laid aside, but the use of the above-mentioned medicine continued. The insolation was repeated twice a-day, for half an hour before, and three-quarters of an hour after, dinner. The patient was not suffered to open his eye-lids directly after the insolation, but only an hour afterwards, and then only in a darkened, though not quite dark, room. After having proceeded thus for a fortnight, he found the iris to have acquired more power of motion, and the patient, at the same time, complained of an itching sensation in the afflicted eye, but could merely discern the motion of the hand before the same. Mr. Loebel continued the insolation, and had the satisfaction of seeing his patient in a short time cured of his amaurosis. He could discern every object, distinguished all his acquaintances that came to visit him: however he could not read any printed or written characters, nor could all Mr. Loebel's art bring his patient so far as to enable him to read a book with his right eye.

From subsequent foreign journals we make the following extract:—

A very ingenious oculist, Prof. Weinhold, M.D. at Merseburg, has published the following remarks in recommendation of the use of Insolation upon the torpid Retina, in the Jena Literary News, Merseburg, Oct. 4, 1815.

"I fully coincide in recommending insolation through *half-convex* glasses, having, in quality of practical physician and oculist, frequent occasions to observe the danger attending the use of the common burning glass. For this reason I commonly cause large burning burning glasses to be cut into halves, or cover them half with black paper, by which means the dangerous focus is avoided.

Insolation proved disadvantageous in the amauroses accompanied by a heightened irritability, but advantageous in that attended with torpor, or, as the ancients say, *sine materia*, of course in the amaurosis the consequence of nervous complaints, unattended with gout, lues venerea, or psora."

Mr. Ludewig, surgeon at Naufung, cured a chronic inflammation of the eyes, that had lasted many years, by vaccination.

In the spasmodic complaints of a woman which did not yield to the usual remedies, cobwebs, made up with mica panis into pills, procured a lasting amendment.

The efficacy of burnt sponge, prepared according to the directions of Dr. Hausleuther, given in Horn's Archive for 1810, is fully confirmed by Dr. Karsten, in a case of a large and obstinate struma.

---

At a meeting of the subscribers for establishing "*The Medical Benevolent Society*," for the relief of medical men who, either from age or adversity, shall require it, held at the Freemason's Tavern, London, January 29th, 1816, Dr. Latham, President of the Royal College of Physicians, in the chair, it was resolved—

"That, as it appears that the time allowed for receiving the names of subscribers has not been sufficient for gentlemen resident in the country to declare their intentions; therefore the books for that purpose shall remain open at Messrs. Child's, Bankers, Temple Bar, and at this Tavern, till February the 24th.

"That, until the 20th of March next, benefactions and subscriptions will be received by any of the Treasurers, viz. Dr. Clutterbuck, New Bridge-street; Richard Ogle, Esq. Great Russel-street, Bloomsbury; and Henry Field, Esq. Christ's Hospital; or by Messrs. Child's the Bankers."

John Latham, Chairman.

Several distinguished characters in the profession, amongst whom Dr. Hull, of Manchester, was conspicuous, sent liberal benefactions, and many subscribers entered their names on the books; and the Society commences under the happiest auspices.

The following is the list of officers for the Society:

President.—Dr. Latham.

Vice-President.—Henry Cline, Esq.

Directors.—Drs. Haworth, Bateman, Merriman, Luke, P. M. Latham, Ridout; Messrs. Burrows, Hayes, A. T. Thomson, C. M. Clarke, Shillito, Lockley, Tegart, E. Brande, R. S. Wells, Malim, P. Mathias, Reginald Williams.

Trustees.—Dr. Latham, Richard Radford, Esq.

Treasurers.—Dr. Clutterbuck, Mr. Ogle, Mr. Field.

### *General Bill of Mortality, from Dec. 13, 1814, to Dec. 12, 1815, as delivered to the King by the Company of Parish Clerks.*

| DISEASES. | |
|---|---|
| Abortive & Stilborn | 804 |
| Abscess | 105 |
| Aged | 1757 |
| Ague | 5 |
| Apoplexy & Suddenly | 421 |
| Asthma | 680 |
| Bedridden | 2 |
| Bile | 5 |
| Bleeding | 23 |
| Bursten & Rupture | 34 |
| Cancer | 88 |
| Chicken-pox | 2 |
| Childbed | 232 |
| Colds | 16 |
| Colic, Gripes, &c. | 26 |
| Consumption | 4210 |
| Convulsions | 3324 |
| Cough and Hooping Cough | 729 |
| Cramp | 4 |
| Croup | 87 |
| Diabetes | 6 |
| Dropsy | 792 |
| Epilepsy | 1 |
| Evil | 7 |
| Fevers of all kinds | 1309 |
| Fistula | 3 |
| Flux | 65 |
| French Pox | 22 |
| Gout | 67 |
| Gravel, Stone, Strangury | 16 |
| Grief | 5 |
| Headmoldshot, Horseshoehead, & Water in the Head | 383 |
| Imposthume | 4 |
| Inflammation | 953 |
| Influenza | 1 |
| Jaundice | 90 |
| Lethargy | 1 |
| Livergrown | 46 |
| Lumbago | 3 |
| Lunatic | 228 |
| Measles | 711 |
| Miscarriage | 2 |
| Mortification | 306 |
| Palpitation of the Heart | 6 |
| Palsy | 163 |
| Pleurisy | 18 |
| Quinsy | 5 |
| Rash | 1 |
| Rheumatism | 9 |
| Scrophula | 3 |
| Scurvy | 4 |
| Small-pox | 725 |
| Sore Throat | 5 |
| Sores and Ulcers | 11 |
| Spasm | 36 |
| St. Anthony's Fire | 9 |
| Stoppage in the Stomach | 23 |
| Surfeit | 1 |
| St. Vitus's Dance | 2 |
| Teeth | 447 |
| Thrush | 118 |
| Tumor | 3 |
| Water in the Chest | 30 |
| Worms | 8 |

| CASUALTIES. | |
|---|---|
| Bit by Dogs | 2 |
| Broken Limbs | 1 |
| Bruised | 2 |
| Burnt | 32 |
| Drowned | 132 |
| Excessive Drinking | 8 |
| Executed | 8 |
| Found Dead | 25 |
| Fractured | 3 |
| Frighted | 5 |
| Killed by Falls & several other Accidents | 76 |
| Killed themselves | 47 |
| Murdered | 1 |
| Overlaid | 1 |
| Poisoned | 1 |
| Scalded | 10 |
| Shot | 1 |
| Suffocated | [illegible] |

Christened { Males 12281, Females 11133 } In all 23414

Buried . { Males 9882, Females 9678 } In all 19560

Whereof have died,

| | |
|---|---|
| Under Two Years of Age | 5200 |
| Between Two and Five | 1916 |
| Five and Ten | 870 |
| Ten and Twenty | 677 |
| Twenty and Thirty | 1425 |
| Thirty and Forty | 1824 |
| Forty and Fifty | 2075 |
| Fifty and Sixty | 1886 |
| Sixty and Seventy | 1621 |
| Seventy and Eighty | 1221 |
| Eighty and Ninety | 674 |
| Ninety and a Hundred | 167 |
| A Hundred | 2 |
| A Hundred and One | 1 |
| A Hundred and Three | 1 |

Decreased in the Burials this Year 223.

There have been Executed in the City of London and County of Surrey 20; of which Number 8 only have been reported to be Buried within the Bills of Mortality.

The most striking articles in the above report are the increase of small-pox, though the general number of deaths has been somewhat less this year. Measles, cough, and fever have each lessened. It may seem particularly worthy of notice that the number of christenings have exceeded the burials in the proportion of nearly a seventh part. This is the more remarkable, because the two most populous and probably healthy parishes, Mary-le-bone and Pancras, are not included in the bills. That the health of the town increases cannot be questioned; but it would be very desirable to come as near correctness as possible. It has been said that the present bills are equal to every thing that is required. How can this be the case, when we have no distinction between scarlet and other fevers, and no authority for any other item than the report of parish old women yclept searchers? It is this consideration which prevents our extending these remarks further. Where we have such flimsy authorities, to what purpose is it to form calculations.

---

*Abstract from the St. Petersburgh Almanack for the year* 1815.

The annual publications of the Holy Sinode (which, however, relate only to individuals of the Russian Greek church) inform us, that, in the year 1812, were born in the whole empire—

| | |
|---|---|
| Males | 663,741 |
| Females | 600,650 |
| | 1,264,391 infants. |

41,756 infants less than in 1811.

In the year 1812 died—

| | |
|---|---|
| Males | 501,386 |
| Females | 469,972 |
| | 971,358 |

34,990 more than in 1811.

In 1812 the increase of population was of 293,033 individuals, and by 76,746 less than 1811.

In 1812 were married 239,073 couples of the protestant Greek church, 39,527 couples less than in 1811, and 90,216 couples less than in 1810.

From the same undoubtful authority we abstract the following curious List of Longevity in 1812, a year which must for ever remain memorable in the history of national calamities.

213,746 infants died before the age of 5 years.

| | |
|---|---|
| 87,047 individuals lived past | 60 |
| 45,735 | 70 |
| 18,685 | 80 |
| 4,932 | 90 |
| 2,288 | 95 |
| 760 | 100 |
| 233 | 105 |
| 106 | 110 |
| 53 | 115 |
| 20 | 120 |
| 5 | 125 |

| | |
|---|---|
| 4....individuals lived past...... | 130 |
| 1 ........................... | 160 |

N.B.—We can assure, from the best authority, that old people preserve wonderfully their faculties in Russia, memory particularly, which makes tradition a very precious repository of local knowledge. As the Russians in general use freely of fermented liquors of all kinds, their health, longevity, and particularly absence of gout, must be, in great measure, attributed to the severity of their fasts, to the frequent use of vapour-baths (which keeps the old people in excellent bodily condition, and the younger in cleanliness), and to their religious resignation.

Of St. Petersburgh we find the following account:—

In 1813 were born—

| | |
|---|---|
| Males .............. | 3,828 |
| Females ............ | 3,730 |
| | 7,558 infants. |

315 infants less than in 1812.

In 1813 died—

| | |
|---|---|
| Males.............. | 10,870 |
| Females............ | 4,114 |
| | 14,984 individuals. |

3,977 individuals more than in 1812.

In the above number of born, 1,089 were Roman Catholics and Protestants, in the ratio of the first to the last as 3 to 14.—958 were bastard, and 53 foundlings; therefore ⅛th illegitimate.

The greatest number of births was in March and April (759 males, 684 females); and the smallest in May and September (515 males, and 555 females).

In the above number of dead, 1384 were Roman Catholics and Protestants, in the proportion of the first to the last as 3 to 14½.

44 died of wounds.
481 of various accidents.
4,097 of typhus (this was the most dangerous disease).
3,488 of colics (the next dangerous, principally for infants).
3,324 of consumption and pulmonary disease.
77 in child-bed.
126 small-pox.

The greatest mortality was in March, viz. died 1,894.
The least ditto in November ................ 677.

The following is the list of Longevity at St. Petersburgh in 1813:

| | |
|---|---|
| 3,775 died as infants before | 5 years. |
| 191 . died at from . | 5 to 10 |
| 391............... | 10 .. 15 |
| 885............... | 15 .. 20 |
| 1,943............. | 20 .. 25 |
| 900............... | 25 .. 30 |

| | | | |
|---|---|---|---|
| 2,071 | died at from | 30 | 35 |
| 770 | | 35 | 40 |
| 1,136 | | 40 | 45 |
| 563 | | 45 | 50 |
| 602 | | 50 | 55 |
| 393 | | 55 | 60 |
| 475 | | 60 | 65 |
| 238 | | 65 | 70 |
| 256 | | 70 | 75 |
| 129 | | 75 | 80 |
| 84 | | 80 | 85 |
| 47 | | 85 | 90 |
| 7 | | 90 | 95 |
| 3 | | 95 | 100 |

In 1813 were married, of the Greek church, 1,099 couples.
........................ Protestants ...... 233
........................ Roman Catholics .. 72

1,404 couples.

49 marriages less than in 1812.

The greatest number took place in February, viz. 202; and the least in March, only 8; and in April only 19.

Out of the above number, 1,068 were bachelors to spinsters.
106 ............... widows.
136 .... widowers to spinsters.
80 ............... widows.
7 .. bachelors to divorced women.
2 .... divorced men to spinsters.
5 divorced men to divorced women.

1,404 couples, as above.

---

*Medical School of St. Thomas's and Guy's Hospitals.*—The Spring Courses of Lectures at these adjoining Hospitals will commence the beginning of February, as follows:

At St. Thomas's—Anatomy and Operations of Surgery, by Mr. Astley Cooper and Mr. H. Cline. Principles and Practice of Surgery, by Mr. Astley Cooper.

At Guy's—Practice of Medicine, by Dr. Babington and Dr. Curry. Chemistry, by Dr. Babington, Dr. Marcet, and Mr. Allen. Experimental Philosophy, by Mr. Allen. Theory of Medicine, and Materia Medica, by Dr. Curry and Dr. Cholmeley. Midwifery, and Diseases of Women and Children, by Dr. Haighton. Physiology, or Laws of the Animal Œconomy, by Dr. Haighton. Structure and Diseases of the Teeth, by Mr. Fox.

---

*Russell Institution.*—A Course of Lectures on Electrical Philosophy, with its application to the improvement of Chemical Science, and and the explanation of Natural Phenomena, will be commenced at this Institution by Mr. SINGER, on Monday the 5th of February, punctually at eight o'clock in the evening.

These Lectures will be continued on the succeeding Mondays at the same hour:—they will embrace the most important features of this interesting branch of Natural Philosophy, with occasional observations on the Sciences with which it is most immediately connected.

---

Dr. MERRIMAN will re-commence his Lectures on Midwifery at the Middlesex Hospital, on Thursday, February 8th, at half-past ten o'clock.

---

Dr. ARMSTRONG, of Sunderland, has in the press, a work entitled, Practical Illustrations of Febrile Diseases. The principal object of this work is to shew the great advantages of early and decided blood-letting and purging in Typhus, in a disease called the Common Inflammatory Fever, in Scarlatina Maligna, in Measles, in Erysipelas, in Hydrocephalus Internus, in Dysentery, and other affections. It will be illustrated with several cases and dissections.

---

Speedily will be published, in two volumes, octavo, the Institutes and Practice of Medicine, founded on the Basis of Anatomy, Healthy and Morbid; and on the well-known Laws of the Animal Economy. By LYMAN SPALDING, M.D. President of the College of Physicians and Surgeons of the University of New York for the Western District.

---

Dr. GRANVILLE has in the press, and nearly ready for publication, a translation of that part of Orfila's general Toxicology which more particularly relates to Poisons derived from the Vegetable and Animal Kingdoms. The subject having formed a very important part of Dr. Granville's scientific pursuits, he has been enabled to accompany his translation with copious notes and additions.—The original has only been before the public a few days, and is not yet in general circulation.

---

In a few days will be published, the second part of Orfila's System of Animal, Mineral, and Vegetable Poisons. This will complete the first volume of this very interesting work. The two remaining parts will appear during the ensuing spring.

---

Shortly will be published, in one octavo volume, an Account of a Visit to London in the Year 1814; wherein is exhibited a comparative View of Surgery in England and in France, preceded with Considerations respecting the London Hospitals: translated from the French of Monsieur Roux, Professor of Anatomy and Surgery at Paris.

METEORO-

### METEOROLOGICAL REGISTER.

***From December the 25th,* 1815, *to January the 26th,* 1816.**

**Kept by C. BLUNT, Philosophical Instrument Maker, No. 38, Tavistock-Street, Covent-Garden.**

| Moon. | Day. | Wind. | Barometrical Pressure. Max. | Min. | Mean. | Temperature. Max. | Min. | Mean. | |
|---|---|---|---|---|---|---|---|---|---|
| | 26 | W | 29 49 | 29·11 | 29 312 | 50 | 28 | 41·25 | Snow&Rain |
| | 27 | NW | 29 69 | 29 07 | 29 375 | 50 | 26 | 39·5 | Snow |
| | 28 | NW | 29·83 | 29 76 | 29·795 | 50 | 28 | 38· | Rain |
| | 29 | W | 29 90 | 29·84 | 29 877 | 49 | 32 | 40·5 | Fair |
| ● | 30 | W | 30·40 | 30·14 | 30·287 | 50 | 30 | 42·75 | Fair |
| | 31 | W | 30·40 | 30·40 | 30·40 | 47 | 32 | 40 75 | Fair |
| | 1 | W | 30 38 | 30·27 | 30·32 | 46 | 30 | 39·25 | Fair |
| | 2 | W | 30·15 | 30·02 | 30·087 | 44 | 32 | 38·5 | Fair |
| | 3 | W | 30·14 | 30· | 30 08 | 44 | 25 | 35 25 | Fair |
| | 4 | W | 30·28 | 30·23 | 30·267 | 43 | 26 | 35· | Fair |
| | 5 | W | 30 12 | 30·08 | 30·095 | 42 | 25 | 34· | Rain |
| | 6 | W | 29·78 | 29·66 | 29 715 | 45 | 32 | 40· | Fair |
| ◐ | 7 | NW | 29 64 | 29·64 | 29·64 | 43 | 33 | 38 5 | Fair |
| | 8 | NW | 29 63 | 29·38 | 29·50 | 48 | 32 | 42· | Rain |
| | 9 | W | 29 50 | 29 46 | 29 382 | 47 | 33 | 42· | Fair |
| | 10 | W | 29 40 | 29·30 | 29 34 | 48 | 35 | 42·5 | Fair |
| | 11 | W | 29·13 | 29 99 | 29 045 | 44 | 39 | 42·5 | Fair |
| | 12 | W | 29·46 | 29·13 | 29 325 | 46 | 35 | 41·75 | Rain |
| | 13 | W | 29 03 | 28·95 | 28·99 | 46 | 36 | 42· | Fair |
| ○ | 14 | SW | 29·22 | 29·22 | 29 22 | 46 | 35 | 42· | Rain |
| | 15 | S | 29·30 | 29·17 | 29·20 | 46 | 34 | 40·5 | Fair |
| | 16 | NW | 29·66 | 29 57 | 29·63 | 47 | 34 | 40 5 | Fair |
| | 17 | NW | 29 60 | 29 46 | 29·545 | 49 | 35 | 40·75 | Fair |
| | 18 | W | 29·65 | 29·65 | 29 65 | 49 | 33 | 41·25 | Rain |
| | 19 | W | 29·77 | 29 70 | 29 747 | 50 | 32 | 42·25 | Rain |
| | 20 | SW | 29·58 | 29·52 | 29 54 | 49 | 32 | 42·5 | Rain |
| ◑ | 21 | S | 29 52 | 29·52 | 29·52 | 49 | 32 | 42 5 | Rain |
| | 22 | S | 29·55 | 29 52 | 29·54 | 50 | 33 | 42·5 | Fair |
| | 23 | N | 29·53 | 29·43 | 29·462 | 51 | 33 | 43 5 | Fair |
| | 24 | NE | 29·29 | 29·22 | 29 247 | 50 | 35 | 43· | Rain |
| | 25 | NE | 29·16 | 29·16 | 29·16 | 50 | 35 | 43·25 | Rain |

RESULTS.

Mean barometrical pressure of the month 29·557
Maximum 30 40, wind at W
Minimum 28·95, ——— W

Mean temperature of the month 46·46 deg.
Maximum 51, wind at N
Minimum 25, ——— W

*Scale exhibiting the prevailing Winds during the Month.*

| N | NE | E | SE | S | SW | W | NW |
|---|---|---|---|---|---|---|---|
| 1 | 2 | 0 | 0 | 3 | 2 | 17 | 6 |

| | Mean barometrical pressure. | Mean temperature. |
|---|---|---|
| From the last quarter on the 23d Dec. to the new moon on the 30th. | 29 571 | 38·214 |
| ——— new moon on the 30th Dec. to the first quarter on the 7th Jan. 1816 | 30·156 | 38·187 |
| ——— first quarter on the 7th, to the full moon on the 14th | 29·288 | 41·617 |
| ——— full moon on the 14th, to the last quarter on the 21st | 29·504 | 40 307 |

REPORT

### REPORT OF DISEASES.

In our Report of Diseases we shall be very short, not so much on account of the want of room, as on account of the sameness of the temperature, and consequently of the diseases.

The acute diseases have considerably lessened, in number, force, and proportion of fatality. They are very similar to our last report, excepting that small-pox has greatly declined. It has not been succeeded by measles. Scarlatina has occurred, but mostly, if not entirely, among new comers to the metropolis, and very young children. Pleurisies continue, but with less violence; and the improved practice of early and free blood-letting has proved a speedy relief to all who applied early.

Rheumatism, and even gout, have been more frequent among all classes. As we suspected, the bandaging system has failed in some cases; but this ought not to supersede its use. In the early stages of rheumatism, bleeding, as well as purging, are very generally successful. In the more advanced, purging seems the only remedy, excepting that in a very few debilitated subjects the bark has been of service, but in all others it seems to do harm. In the very advanced stage of gout and rheumatism, in which the pains are wandering, and the general irritability excessive, decoction of bark with turpentine has been found serviceable. Do not the virtues of guaiacum depend on its resinous properties, like the turpentine? When the pains are local, nothing is so sure of success as topical bleeding. Where cupping-glasses can be applied, they are greatly preferable to leeches.

